# Overlapping Phenotypes of Alcoholic Cardiomyopathy and Left Ventricular Non-compaction: A Case Report and Discussion of Converging Cardiomyopathies

**DOI:** 10.7759/cureus.48220

**Published:** 2023-11-03

**Authors:** Vaidehi Mendpara, Jaya Krishna Reddy Endreddy, Sahini Gajula, Pratyusha Ravulapalli, Mahendra Kumar, Parvinder Kaur, Meet Thakkar

**Affiliations:** 1 Internal Medicine, Government Medical College, Surat, Surat, IND; 2 Internal Medicine, Kamineni Academy of Medical Sciences and Research Centre, Hyderabad, IND; 3 Internal Medicine, Gandhi Medical College and Hospital, Secunderabad, IND; 4 Internal Medicine, Apollo Institute of Medical Sciences and Research, Hyderabad, IND; 5 Medicine, Sardar Patel Medical College, Bikaner, IND; 6 Internal Medicine, Crimean State Medical University, Simferopol, UKR; 7 General Medicine, Government Medical College, Surat, Surat, IND

**Keywords:** echocardiography, cardiac mri (cmr), dilated cardiomyopathy, endomyocardial trabeculations, congenital cardiomyopathy

## Abstract

Left ventricular non-compaction cardiomyopathy, often known as LVNC, is a form of congenital cardiomyopathy that is extremely uncommon. It is a condition that may be identified by an elevated number of endomyocardial trabeculations as well as an increase in their prominence. Alcoholic cardiomyopathy, also known as ACM, is a non-ischemic form of dilated cardiomyopathy that is characterized by contractile failure and an enlargement of the heart ventricles. It is not entirely known whether or not there is a clinically significant overlap in phenotypic characteristics between the two illnesses. We report a patient who had previously been diagnosed with ACM and who had cardiac MRI results that fit the criteria for both LVNC and ACM.

## Introduction

A relatively uncommon and intriguing heart condition known as left ventricular non-compaction cardiomyopathy (LVNC) is characterized by an abundance of trabeculations and deep recesses in the left ventricular myocardium. Due to the condition's diverse clinical presentation and similarities to other cardiomyopathies, diagnosing it can be difficult. From childhood to maturity, LVNC can affect anyone and present with a variety of symptoms, from minor fatigue to potentially fatal heart failure and arrhythmias [1,2]. Alcoholic cardiomyopathy (ACM), on the other hand, is a condition resulting from chronic alcohol abuse that leads to heart muscle damage. Both conditions can lead to heart failure symptoms and other complications, but they have distinct underlying causes and mechanisms [3].

In this case report, we provide a thorough examination of a patient identified as having left ventricular non-compaction cardiomyopathy and the effect of alcohol on the heart. We hope that this study will give readers a thorough grasp of the clinical characteristics, diagnostic process, management techniques, and results related to this complex cardiomyopathy. We aim to contribute by looking into the complexities of this case.

This article was previously posted to the Authorea preprint server on August 24, 2023.

## Case presentation

**Figure 1:**
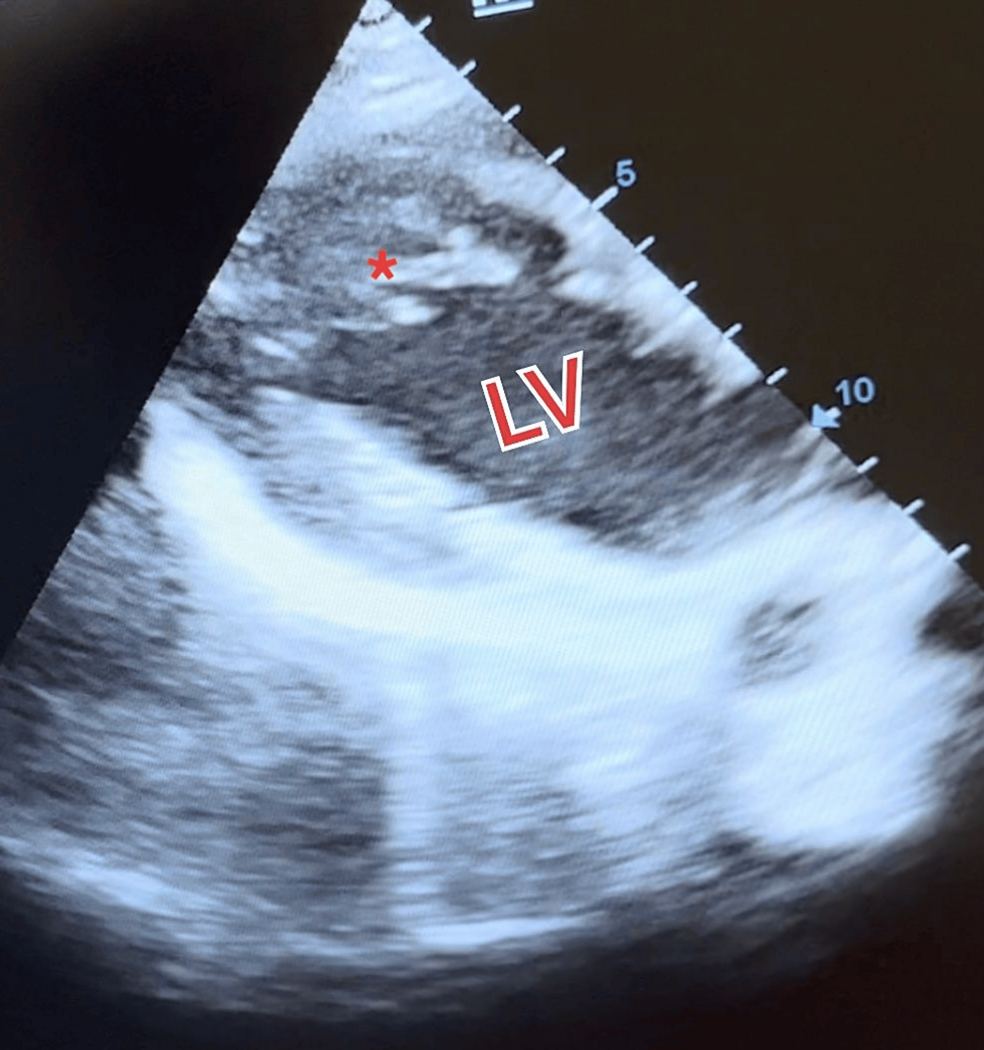
Transthoracic echocardiogram demonstrating left ventricular non-compacted myocardium and trabeculations (red asterisks)

**Figure 2:**
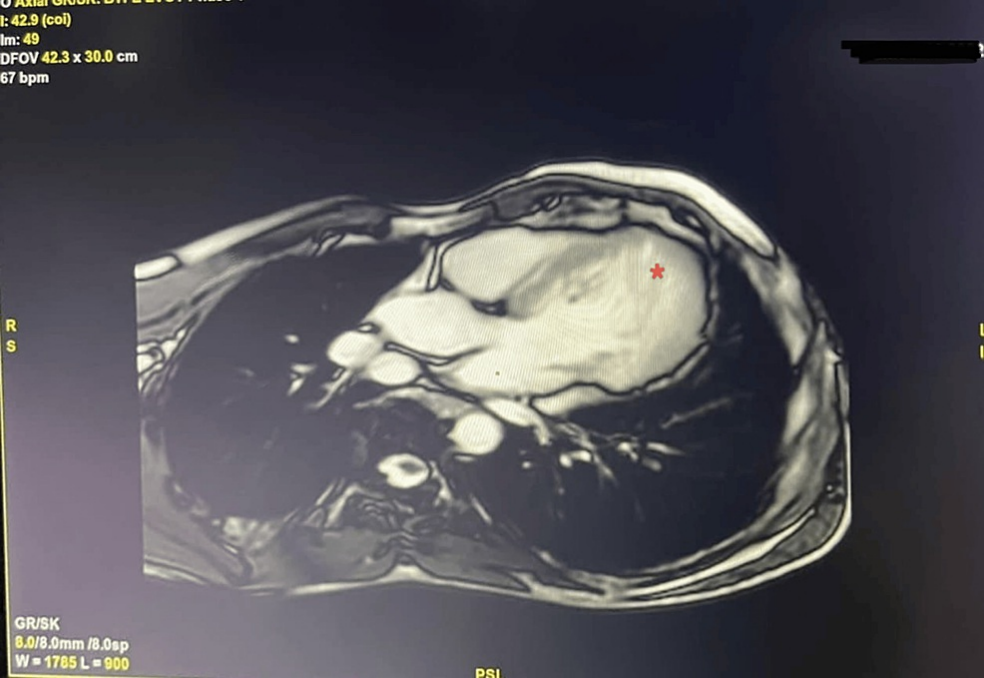
Cardiac MRI demonstrating left ventricular non-compacted myocardium and trabeculations (red asterisks)

In this case, a 49-year-old man with a longstanding history of chronic alcohol consumption presented at the outpatient clinic with a constellation of symptoms including dyspnea, chest pain, and palpitations. A physical examination revealed bilateral lung crackles and lower extremity edema, prompting further investigation. The first 2D echocardiogram showed signs of ACM, like global hypokinesia and a reduced left ventricular ejection fraction (LVEF) of 35%. His echocardiogram incidentally revealed prominent left ventricular trabeculations, raising suspicions of left ventricular noncompaction cardiomyopathy (Figure 1). Subsequent cardiac magnetic resonance imaging (MRI) confirmed both diagnoses, showcasing hypertrabeculation of the left ventricle with a > 2:1 ratio of non-compacted to compact myocardium while also reiterating the alcoholic cardiomyopathy (ACM) characteristics of ventricular dilation and contractile dysfunction (Figure 2). The cardiac MRI revealed the presence of non-compacted myocardium and trabeculations in the left ventricle, occurring at various levels (Video 1).

**Video 1:** Cardiac MRI demonstrating left ventricular non-compacted myocardium and trabeculations

A comprehensive treatment strategy was initiated, encompassing aspirin, sacubitril-valsartan, furosemide, metoprolol, atorvastatin, and spironolactone, supplemented by strong counseling for alcohol abstinence. Encouragingly, the patient responded positively to treatment, marked by a regression of symptoms and improved exercise capacity. Scheduled follow-ups with the cardiologist are planned for ongoing arrhythmia screening and monitoring of left ventricular function.

## Discussion

LVNC is a newly recognized congenital heart condition characterized by a "spongy" appearance of the myocardium, primarily at the left ventricular apex; hence, it is also named spongy myocardium, spongiform cardiomyopathy, hypertrabeculation, and persisting myocardial sinusoids. It can be associated with complex congenital heart defects or skeletal myopathy. It represents a halt in the normal maturation of the myocardium. This disease can present throughout life with progressive left ventricular systolic dysfunction [4]. It results from disrupted embryonic development, leading to prominent trabeculae and deep intertrabecular recesses, creating a two-layered left ventricular myocardium with a thin epicardium and thickened endocardial layers [5]. LVNC has been proven to be a genetically heterogeneous cardiomyopathy, and it appears to arise from abnormal developmental processes [6]. Left ventricular non-compaction is within the diverse spectrum of cardiac morphologies triggered by sarcomere protein gene defects [7]. It can be associated with left ventricular dilation or hypertrophy, systolic or diastolic dysfunction, or various forms of congenital heart disease. While its natural history is not fully understood, it may lead to reduced LV function, heart failure, blood clot formation, irregular heart rhythms, sudden death, and cardiac remodeling [8].

A review of 59 studies in patients aged 12 and older found that the prevalence of LVNC differed depending on the diagnostic method used. Echocardiography showed a prevalence of 1.28%, while cardiac magnetic resonance imaging indicated a higher prevalence of 14.79%. Athletes had higher rates (3.16% by echocardiography and 27.92% by cardiac MRI). These variations highlight the challenges in assessing LVNC prevalence due to differing diagnostic criteria and population differences [9]. ACM, which represents 3.8% of all cardiomyopathy cases, has a variable prevalence ranging from 23% to 40%. It involves increased heart muscle mass, ventricle dilation, and thinner walls. Ventricular function varies with the disease stage, with asymptomatic ACM linked to impaired relaxation and symptomatic ACM often showing reduced heart contraction [10]. Our patient presented clinical features of heart failure with diagnostic evidence of both conditions, LVNC and ACM.

The preferred diagnostic procedure is two-dimensional echocardiography. It can identify a two-layer myocardium with trabeculations and deep intertrabecular recesses that is composed of a thick non-compacted layer and a thin compacted layer at the mid-ventricular and apical levels. For the diagnosis of LVNC, there are three sets of echocardiographic criteria. The most commonly acknowledged verified echocardiographic criteria are the Jenni criteria, also known as Zurich criteria, for LVNC. They are evaluated at the base, mid, and apical levels in the parasternal short-axis view [11]. As an alternative, some doctors employ the similarly verified Chin or Stöllberger criteria. According to the Chin, or California, criterion [12], LVNC is defined as the ratio X-to-Y less than or equal to 0.5, where X is the distance between the epicardium and the trabecular trough and Y is the distance between the epicardium and the trabecular peak. On subxiphoid or apical four-chamber views at the end of diastole, this criterion is applied to trabeculae at the LV apex. The Vienna criteria, also known as the Stöllberger criteria [13], place a strong emphasis on hypertrabeculation.

Cardiac MRI (CMRI) imaging aids in more accurate LVNC identification. Assessment of the non-compacted to compacted ratio is one of the CMR criteria by Peterson with a sensitivity of 86% and specificity of 99%, which must be greater than 2.3 during diastole in any long-axis LV image to make a diagnosis of LVNC [14]. A highly sensitive and precise criterion was suggested to be used by Jacquier et al. [15]. A trabeculated left ventricular mass should be greater than 20% of the total LV mass. Using late gadolinium enhancement, cardiac magnetic resonance may also measure the degree of heart fibrosis, which is connected to negative clinical outcomes. A role for three-dimensional echocardiography has not been established. Additionally, a role for contrast echocardiography has been incompletely evaluated in LVNC. Case studies that use cardiac computed tomography (CT) to diagnose LVNC have been published in the literature [16]. With cardiac CT, LVNC may be identified with high spatial resolution [16,17].

The objectives of treatment include symptom management and lowering the risk of complications. Digoxin, diuretics, ACE inhibitors, beta-blockers, and afterload reduction medications are used to treat symptomatic patients. Anticoagulation and primary prevention of sudden cardiac death (SCD) are two management concerns that are specific to LVNC patients. If a left ventricular clot has been seen on imaging or if the patient has been diagnosed with atrial fibrillation, oral anticoagulation should be taken. CHADS2/CHADS2-Vasc scores should be used as guidance for risk assessment for patients who do not fit into either of these groups. Ejection fraction (EF) and degree of symptoms can help define goals of management in terms of the placement of an implantable cardioverter-defibrillator (ICD). It makes sense to move on with this technique if the patient satisfies the ICD criteria based on reduced EF. It has been documented that with normal EF, patients still have an increased chance of developing SCD [18]. When a patient has a normal EF but a history of syncope, nonsustained ventricular tachycardia, or a family history of SCD, the doctor should discuss the advantages and disadvantages of ICD therapy with the patient as a possible course of treatment [19]. Besides these, cardiac transplantation may help some individuals.

Left ventricular non-compaction is a rare congenital cardiomyopathy. Patients diagnosed with diabetes often have a poor prognosis, predisposing them to cardiac failure, sepsis, and cerebrovascular events. Due to the disorder's rarity and varied presentation, the best way to address it is with a team of professionals.

## Conclusions

This case report underscores the diagnostic challenges posed by concurrent LVNC and ACM. The patient's chronic alcohol use led to ACM with ventricular dilation, while LVNC indicators like elevated trabeculations were also present. Echocardiography and cardiac MRI confirmed both diagnoses. The case emphasizes the need for accurate diagnostic techniques and a comprehensive approach to differentiate overlapping cardiomyopathies. Successful management involved diverse interventions and counseling. The positive treatment response highlights the significance of early diagnosis. This report showcases the intricate interplay of symptoms, imaging, and diagnostic criteria in understanding complex cardiomyopathies for improved patient care.

## References

[1] (2023). Left Ventricular Non-Compaction Cardiomyopathy (LVNC) | Pediatric Cardiomyopathy. Left Ventricular Non-Compaction Cardiomyopathy (LVNC) | Pediatric Cardiomyopathy. https://www.cincinnatichildrens.org/service/c/cardiomyopathy/types/left-ventricular-non-compaction-cardiomyopathy.

[2] Towbin JA, Jefferies JL (2017). Cardiomyopathies due to left ventricular noncompaction, mitochondrial and storage diseases, and inborn errors of metabolism. Circ Res.

[3] Shaaban A, Gangwani MK, Pendela VS (2023). Alcoholic cardiomyopathy. StatPearls [Internet].

[4] Singh DP, Patel H (2023). Left ventricular noncompaction cardiomyopathy. https://www.ncbi.nlm.nih.gov/books/NBK537025/.

[5] Maron BJ, Towbin JA, Thiene G (2006). Contemporary definitions and classification of the cardiomyopathies: an American Heart Association Scientific Statement from the Council on Clinical Cardiology, Heart Failure and Transplantation Committee; Quality of Care and Outcomes Research and Functional Genomics and Translational Biology Interdisciplinary Working Groups; and Council on Epidemiology and Prevention. Circulation.

[6] Rojanasopondist P, Nesheiwat L, Piombo S, Porter GA Jr, Ren M, Phoon CK (2022). Genetic basis of left ventricular noncompaction. Circ Genom Precis Med.

[7] Klaassen S, Probst S, Oechslin E (2008). Mutations in sarcomere protein genes in left ventricular noncompaction. Circulation.

[8] Towbin JA, Lorts A, Jefferies JL (386). Left ventricular non-compaction cardiomyopathy. Lancet (London).

[9] Ross SB, Jones K, Blanch B, Puranik R, McGeechan K, Barratt A, Semsarian C (2020). A systematic review and meta-analysis of the prevalence of left ventricular non-compaction in adults. Eur Heart J.

[10] Piano MR (2002). Alcoholic cardiomyopathy: incidence, clinical characteristics, and pathophysiology. Chest.

[11] Jenni R, Oechslin E, Schneider J, Attenhofer Jost C, Kaufmann PA (2001). Echocardiographic and pathoanatomical characteristics of isolated left ventricular non-compaction: a step towards classification as a distinct cardiomyopathy. Heart.

[12] Chin TK, Perloff JK, Williams RG, Jue K, Mohrmann R (1990). Isolated noncompaction of left ventricular myocardium. A study of eight cases. Circulation.

[13] Stöllberger C, Gerecke B, Engberding R (2015). Interobserver agreement of the echocardiographic diagnosis of LV hypertrabeculation/noncompaction. JACC Cardiovasc Imaging.

[14] Oryshchyn N, Ivaniv Y (2019242023). Left ventricular non-compaction cardiomyopathy. Proc Shevchenko Sci Soc Med Sci [Internet.

[15] Jacquier A, Thuny F, Jop B (2010). Measurement of trabeculated left ventricular mass using cardiac magnetic resonance imaging in the diagnosis of left ventricular non-compaction. Eur Heart J.

[16] Benjamin MM, Khetan RA, Kowal RC, Schussler JM (2012). Diagnosis of left ventricular noncompaction by computed tomography. Proc (Bayl Univ Med Cent).

[17] Gandhi RT, Sarraf G, Budoff M (2010). Isolated noncompaction of the left ventricular myocardium diagnosed upon cardiovascular multidetector computed tomography. Tex Heart Inst J.

[18] Caliskan K, Szili-Torok T, Theuns DA (2011). Indications and outcome of implantable cardioverter-defibrillators for primary and secondary prophylaxis in patients with noncompaction cardiomyopathy. J Cardiovasc Electrophysiol.

[19] Bennett CE, Freudenberger R (2016). The current approach to diagnosis and management of left ventricular noncompaction cardiomyopathy: review of the literature. Cardiol Res Pract.

